# Supplementary material for: Symmetry broken spin reorientation transition in epitaxial MgO/Fe/MgO layers with competing anisotropies
Source: Sci Rep. 2018 Jun 21;8:9463. doi: 10.1038/s41598-018-27720-7 (PMC6013435; doi:10.1038/s41598-018-27720-7)
Supplement: Supplementary file 1 — Supplementary material [file 41598_2018_27720_MOESM1_ESM.pdf]

Symmetry broken spin reorientation transition in epitaxial MgO/Fe/MgO layers with competing anisotropies

Isidoro Martínez,[1] Coriolan Tiusan,[2] Michel Hehn,[3] Mairbek Chshiev,[4] and Farkhad G. Aliev[1]

[1] *Dpto. Fisica de la Materia Condensada, IFIMAC and INC, Universidad Autonoma de Madrid, 28049, Madrid, Spain*
[2] *Center of Superconductivity, Spintronics and Surface Science (C4S), Technical University of Cluj-Napoca, 400114 Romania*
[3] *Institut Jean Lamour, Nancy-Université Vandoeuvre Les Nancy Cedex, 54506 France*
[4] *Université Grenoble Alpes, CEA, CNRS, INAC-SPINTEC, 38000, Grenoble, France*

This part describes Supplemental Materials

I. EXPERIMENTS

A. Influence of the magnetic sweep history on the magnetization reversal when the field is applied perpendicular

We have verified if the field dependent TMR remains asymmetric when one inverts the direction of the sweep of perpendicular magnetic field. Supplemental Figure 1a,b shows, for the MTJ different to the one represented in the main text, that the existence of the in-plane to the out of plane transition asymmetry is unaffected by the magnetic field history when the perpendicular magnetic field is swept with a 3kOe range.

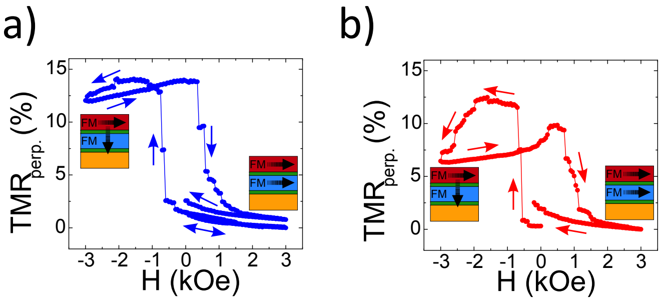

FIG. 1. TMR vs. perpendicular to the plane magnetic field measured at T=5K. Two different magnetic field sweeps (indicated by blue and red arrows) were made as follows: a) 0 → +3kOe → -3kOe → +3kOe → 0 Oe and b) 0 → -3kOe → +3kOe → -3kOe → 0 Oe. The black arrows indicate the magnetization's orientation in the FM electrodes.

II. SIMULATIONS

A. Influence of the cubic anisotropy on the spin reorientation transition

Our simulations shown in supplemental Figure 2 demonstrate that the presence of cubic anisotropy K_c in the Fe film has only a negligible effect on the main parameters of the spin reorientation transition. This fact indicates that the in-plane ground state configuration is mainly formed due to the demagnetization field.

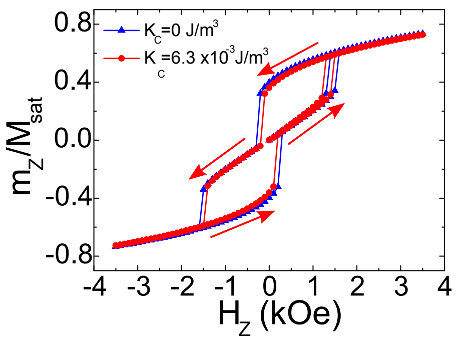

FIG. 2. Spin reorientation transition simulated for the Fe layer with and without cubic anisotropy K_c. The simulations have been done for a symmetric MgO/Fe/MgO structure within model $M1$ and using a PMA value of $K_{s1} = 6.3 \times 10^{-3}$J/m^2. The red arrows indicate the magnetic field sweep history.

B. Influence of the coupling between soft and hard layers on the spin reorientation transition

Our simulations have shown that a ferromagnetic or antiferromagnetic coupling of about 1×10^{-2}J/m^2 between the free and the sensing layers (hard layer) may affect spin reorientation transition. Figure 3 shows that the introduction of the coupling tends to suppress the in-plane to out of plane reorientation transition type in comparison with the uncoupled case discussed in the main text. These simulation results indicate that our sensing hard layer is almost uncoupled to the free layer under study.

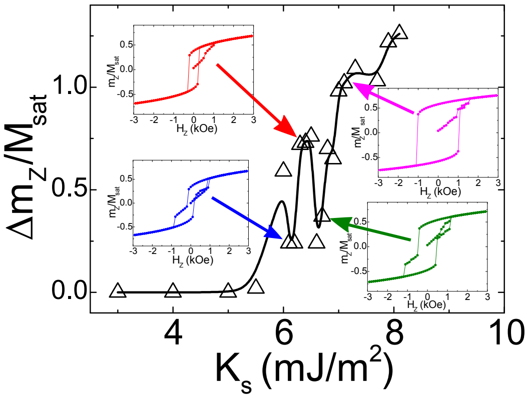

FIG. 3. Variation of the magnetization jump on PMA in 10nm thick soft Fe layer, represented by the model $M1$ with the MgO/Fe/MgO layers antiferromagnetically coupled to the hard Fe/Co layers with energy density of 1×10^{-2}J/m^2. The insets show some representative hysteresis loops obtained upon increasing the PMA value.
